# Genome-Wide Identification of Vascular Plant One-Zinc-Finger Gene Family in Six Cucurbitaceae Species and the Role of *CmoVOZ2* in Salt and Drought Stress Tolerance

**DOI:** 10.3390/genes15030307

**Published:** 2024-02-27

**Authors:** Minyan Xu, Zhi Zhang, Yuhuan Jiao, Yaling Tu, Xin Zhang

**Affiliations:** National Engineering Laboratory of Crop Stress Resistance Breeding, School of Life Sciences, Anhui Agricultural University, Hefei 230036, China

**Keywords:** transcription factor, *CmoVOZ2*, salt, drought, abiotic stresses, ABA

## Abstract

As a plant-specific transcription factor, the vascular plant one-zinc-finger (VOZ) plays a crucial role in regulating various biological processes. In this study, a total of 17 *VOZ* genes in the Cucurbitaceae family were investigated using various bioinformatics methods. The 17 *VOZ* genes in Cucurbitaceae are distributed across 16 chromosomes. Based on the affinity of VOZ proteins to AtVOZ proteins, these 17 proteins were categorized into two groups: group I encompassed eight VOZ members, while group II comprised nine VOZ members. The expression profiles of CmoVOZs under various hormonal and abiotic stresses indicated that these genes were induced differentially by JA, ABA, GA, salt, and drought stress. Subsequently, CmoVOZ1 and CmoVOZ2 were found to be transcriptionally active, with the CmoVOZ2 protein being located mainly in the nucleus. Further experiments revealed that yeast cells expressing *CmoVOZ2* gene showed increased tolerance to salt stress and drought stress. These results suggest that the *VOZ* gene family is not only important for plant growth and development but also that this mechanism may be universal across yeast and plants.

## 1. Introduction

Abiotic and biotic stresses pose significant challenges to plant growth, leading to reduced crop yield and compromised quality [1,2]. In response to these challenges, plants have developed intricate molecular mechanisms to adapt, prompting the exploration and analysis of numerous relevant genes [3,4]. Transcription factors, acting as pivotal molecular switches that regulate the expression of stress-responsive genes, play indispensable roles in the ability of plants to withstand diverse abiotic stresses. Therefore, they are considered promising candidates for genetic enhancement efforts [5,6,7]. Currently, the plant kingdom boasts more than 60 transcription factor families [8]. The vascular plant one-zinc-finger (VOZ) transcription factor family stands out as plant-specific, found exclusively in numerous higher plants, encompassing *Physcomitrella patens* and vascular plants [9,10]. VOZ was identified as a multifunctional gene that regulated various biological processes, including flower induction, development, and responses to diverse stresses [11,12,13,14]. Initially identified in *Arabidopsis thaliana*, this gene family comprised two members, namely *AtVOZ1* and *AtVOZ2*, which both demonstrated binding affinity to the GCGTNx7ACGC palindromic sequence during in vitro studies [9]. The VOZ proteins feature two conserved domains, denoted as A and B. The B domain, commonly referred to as the VOZ domain, comprises a basic region and a zinc finger motif. Subsequent investigations have indicated that the VOZ domain may facilitate the DNA domain’s dimerization across all VOZ proteins [9].

Within the rice genome, two *VOZ* transcription factor genes have been identified. Studies indicate that the *OsVOZ1* gene exhibits expression in various tissues, including roots, stems, and leaves, and its expression is induced under diverse stress conditions. Furthermore, the overexpression of the *OsVOZ1* gene enhances the salt stress resistance of transgenic rice plants, and *VOZ1* and *VOZ2* genes regulate arsenic tolerance and distribution in rice [15]. In the soybean genome, the identification of six *VOZ* genes has been documented. Under conditions of salicylic acid (SA), salt, and dehydration stress, distinct expression patterns are observed in *GmVOZs*. Notably, *GmVOZ1G* exhibits significantly induced expression across all stress treatments. The overexpression of the *GmVOZ1G* gene in soybeans enhanced tolerance to salt and drought stresses. Conversely, the RNA interference (RNAi)-mediated suppression of *GmVOZ1G* expression in soybean hairy roots results in heightened sensitivity to these stress conditions. The findings strongly suggest a positive regulatory role for the *GmVOZ1G* gene in responding to salt and drought stresses in soybean hairy roots [11]. In *Arabidopsis*, *AtVOZ1* exhibits a distinct expression profile with pronounced localization in the phloem, while *AtVOZ2* is notably more abundant in the roots [9]. Moreover, *AtVOZs* engage in interactions with phytochrome B, thereby governing the transition from vegetative growth to flowering through the regulation of FLOWERING LOCUS C (FLC) and FLOWERING LOCUS T (FT) expression. Subsequent studies have shown that the *voz1voz2* double mutant in *Arabidopsis* exhibits heightened tolerance to drought and cold stress but diminished resistance to heat stress and pathogens [16,17]. Additionally, both *AtVOZ1* and *AtVOZ2* have been validated for their capacity to augment salt tolerance, potentially attributable to their direct or indirect regulatory influence on the transcriptional levels of diverse salt-responsive genes [14]. In quinoa (*Chenopodium quinoa*), four *CqVOZ* genes were identified with a high expression in inflorescences and relatively low expression in leaves and stems, and these genes responded to abiotic stresses, including cold stress, salt stress, and drought stress [12]. In *Solanum lycopersicum*, VOZ transcription factors are involved in the tomato fruit ripening process and its response to salt stress [13]. These findings collectively underscore the pivotal contribution of VOZ transcription factors to the intricate regulation of plant growth, particularly in enhancing stress tolerance [18,19,20]. Consequently, the identification and characterization of novel *VOZ* genes across various plant species offer valuable insights into the broader landscape of the *VOZ* gene family. We summarized the functions of the different members of the *VOZ* gene family reported to date in different taxa and tissues (Table S1).

Cucurbits are extensively distributed in tropical and subtropical regions [21]. These plants are primarily valued for their fruits, with examples such as watermelon and cucumber, which are consumed as fresh fruits [22,23,24]. Many cucurbits boast essential nutrients, for instance, watermelon contains sugar and lycopene [25], while wax gourd provides flavonoids and vitamins [26]. Notably, metabolites derived from cucurbit crops have medicinal applications, including amino acids. Additionally, cucurbitacins produced by Cucurbitaceae play a significant role in cancer treatment [27,28]. Studies on VOZ transcription factors focused on *A. thaliana* [14,16], on rice [15] and soybean [11], while studies on cucurbits are limited. Recognizing the crucial role of *VOZ* genes in floral organ development and plant adaptation to adversity, we systematically curated and analyzed published data on *VOZ* genes in cucurbits using bioinformatics. The outcomes are anticipated to provide valuable references for the cloning and functional studies of VOZ transcription factors in cucurbits.

In this study, we identified and characterized the *VOZ* gene family within the Cucurbitaceae. Subsequently, a systematic analysis encompassing gene structure, chromosomal location, conserved motifs, phylogenetic relationships, and *cis*-acting elements was conducted for this gene family. Furthermore, an exploration of the transcriptional activity of the *VOZ* gene family in pumpkin was undertaken, with a specific focus on elucidating the function of the *VOZ2* gene under conditions of salt and drought stress. The findings of this study are expected to offer valuable insights into the functional delineation of *VOZ* genes within the Cucurbitaceae.

## 2. Materials and Methods

### 2.1. Identification of VOZ Proteins in Cucurbitaceae and Evolutionary Analysis

The protein sequences for six species, namely *Citrullus lanatus* (Cla, diploid, 2*n* = 22), *Cucurbita argyrosperma* (Carg, diploid, 2*n* = 20), *Cucumis melo* (Cm, diploid, 2*n* = 24), *Cucurbita moschata* (Cmo, allotetraploid, 2*n* = 40), *Cucumis sativus* (Cs, diploid, 2*n* = 14), and *Lagenaria siceraria* (Lsi, diploid, 2*n* = 22), were retrieved from the Cucurbit Genomics Database (CuGenDB, http://cucurbitgenomics.org/, accessed on 15 November 2023). AtVOZ proteins were obtained from the phytozome database (https://phytozome-next.jgi.doe.gov/, accessed on 17 November 2023) for reference [29]. VOZ protein sequences were acquired using the BLASTP program, utilizing the two *Arabidopsis* VOZ protein sequences as query sequences. Candidate sequences’ conserved domains were annotated using the Pfamscan (https://www.ebi.ac.uk/Tools/pfa/pfamscan/, accessed on 19 November 2023) [30]. A comparison of VOZ proteins from seven species was performed using the ClustalW program in the MEGA11 software (v11). Phylogenetic analysis was conducted through the neighbor-joining method, employing the self-help method of phylogenetic experiments (Bootstrap = 1000, p-distance model) within MEGA11 software (v11) [31].

### 2.2. Chromosomal Localization Analysis and Physicochemical Properties

The coding sequence (CDS), length, and chromosomal location information of the *VOZ* genes were obtained from the Cucurbitaceae website and subsequently visualized through TBtools software (v2.061). Details regarding *VOZ* genes, encompassing genome sequences, CDSs, location coordinates, and open reading frame lengths, were retrieved from the CuGenDB Database. The determination of physicochemical properties for VOZ proteins, including relative molecular mass (Mw) and isoelectric point (pI), was performed via predictions using the ExPASy website (https://web.expasy.org/protparam/, accessed on 23 November 2023) [32].

### 2.3. Conserved Motifs, Gene Structure, and Cis-Acting Elements Analysis

To scrutinize the genetic structure of VOZ, gff3 files corresponding to six distinct species were procured from the CuGenDB database. The analysis and identification of conserved motifs within the VOZ amino acid sequences were conducted using the MEME website (https://meme-suite.org/meme/tools/meme, accessed on 24 November 2023), specifying the number of conserved domains as 10 [33]. The prediction of *cis*-acting elements within the 2 Kbp sequence upstream of *VOZ* genes was carried out using the PlantCARE database website (https://bioinformatics.psb.ugent.be/webtools/plantcare/html/, accessed on 28 November 2023) [34]. Subsequently, the acquired results were visualized utilizing TBtools software (v2.061) [35].

### 2.4. Plant Materials and Abiotic Stress Treatment

In this study, pumpkin plants were cultivated in Hoagland’s nutrient solution at a greenhouse under controlled conditions (28 °C, 16 h light/8 h dark, 70–80% humidity). Upon attaining the three-leaf stage, the seedlings underwent three treatments as described in [36]: ① leaves of hormone treatment groups (100 µM ABA, MeJA, and GA) for 0, 3, 6, and 9 h were collected separately for RNA extraction; ② leaves were exposed to drought (20% PEG6000, Solarbio, Beijing, China) for 6 h; and ③ leaves were exposed to salt (150 mM NaCl, Solarbio, Beijing, China) for 6 h to evaluate separately the responsiveness of *CmoVOZ* genes to abiotic stresses. Three biological replicates were set up for each sample. Subsequently, all samples were promptly frozen in liquid nitrogen and stored at −80 °C to ensure their preservation for subsequent analysis.

### 2.5. RNA Extraction and Quantitative Real-Time PCR

Total RNA extraction from the samples was carried out utilizing Trizol reagent (Takara, Beijing, China) following the manufacturer’s specified instructions. Subsequently, first-strand cDNA was synthesized through the reverse transcription of the RNA (1 µg), employing a First-strand cDNA Synthesis Kit (Vazyme, Nanjing, China). The expression level of the *CmoVOZ* gene was determined using quantitative real-time PCR (qRT-PCR) with primers (Table S2). The reaction mixture consisted of 5 µL SYBR green master mix (Vazyme, Nanjing, China), 0.25 µM upstream and downstream primers, 1 µL cDNA, and up to 10 µL with ddH_2_O. The qRT-PCR process involved an initial denaturation step at 95 °C for 30 s, followed by 40 amplification cycles (comprising 95 °C for 10 s and 60 °C for 30 s), a melting curve step at 95 °C for 15 s, and 60 °C for 1 min. Each sample, comprising three biological replicates, was quantified using the 2^−∆∆Ct^ method, relying on the cycling thresholds (Ct) [37], and employing β-actin as an internal control. Three biological replicates and three experimental replicates were performed for each sample.

### 2.6. Transcriptional Activation Analysis and Subcellular Localization

The *CmoVOZs* were amplified from *C. moschata* leaves utilizing their designated primers (Table S2). After amplification, the resultant fragments were ligated into the TA/Blunt-Zero Cloning Vector (Vazyme, Nanjing, China) for subsequent sequencing. To facilitate transcriptional activity and subcellular localization, the coding sequence of the CmoVOZ gene, excluding termination codons, was amplified employing primers detailed in Table S1. The validated sequence was then incorporated into the pCAMBIA1305 vector (utilizing *SpeI* and *XbaI* restriction sites) and the pGBKT7 vector (employing *BamHI* and *EcoRI* restriction sites), both of which were equipped with the Green Fluorescent Protein (GFP) tags. This integration was achieved through homologous recombination techniques, resulting in the generation of recombinant plasmids.

In transient expression investigations, *Nicotiana benthamiana* served as the experimental host. A volume of 2 mL of resuspended *Agrobacterium* strain, harboring both the GFP and 2 mL of resuspended *Agrobacterium* strains carrying CmoVOZ-GFP, was introduced into leaves of 4-week-old *N. benthamiana* plants, respectively. After a 2-day dark infiltration period, the emitted green fluorescence from the VOZ protein was examined at 488 nm, utilizing a confocal microscope (Zeiss, Jena, Germany). For the analysis of transcriptional activity, the pGBKT7 and four distinct CmoVOZ-pGBKT7 constructs were separately introduced into Y1HGold yeast cells. The assessment of their transcriptional activities involved monitoring their growth on selective media, namely SD/-Trp, SD/-Trp-His, and SD/-Trp-His supplemented with X-α-Gal [38].

### 2.7. Drought and Salt Stress Tolerance Analysis of ComVOZ2 in Yeast

The recombinant plasmid (pYES2-CmoVOZ2) obtained by inserting the *ComVOZ2* gene was cloned into the corresponding sites (*BamHI* and *EcoRI*) of the yeast expression vector (pYES2-NT B). Then, pYES2-NT B and pYES2-CmoVOZ2 were transformed into yeast strain INVSC1, respectively [39]. The transformed yeast strains were cultivated in SG-Ura liquid medium at 28 °C on the shaker for one day, facilitating the induction of *CmoVOZ2* expression. For the assessment of yeast cell growth under stress conditions, the collected yeast cells were standardized to uniform cell densities and subjected to treatments with 2.0 M sorbitol and 1.0 M NaCl, respectively, for 6 h. Then, 2.0 μL of yeast cells was spot-dropped onto SG-Ura solid medium spiked with 2.0 M sorbitol and 1.0 M NaCl, respectively, and incubated for 4−5 days at 28 °C. Yeast cell growth was subsequently evaluated based on the observed phenotypic characteristics. In addition, to assess the growth status of yeast cells after stresses, yeast cells were adjusted to the same cell density and then incubated in sorbitol (0.8, 1.2, 1.6, and 2.0 M) and salt solutions (0.5, 1.0, 2.0, and 3.0 M NaCl) at 28 °C, and the optical density (OD_600_) of the yeast cultures was measured using a PerkinElmer EnSpire (Waltham, MA, USA) after 24 h.

## 3. Results

### 3.1. Identification and Evolutionary Analysis of VOZ Gene Family Members in Cucurbitaceae

In this study, six Cucurbitaceae species were selected for comprehensive analysis: winter squash (*C. moschata*), cucumber (*C. sativus*), melon (*C. melo*), bottle gourd (*L. siceraria*), silver-seed gourd (*C. argyrosperma*), and watermelon (*C. lanatus*). A total of 17 VOZ proteins were discerned within the Cucurbitaceae family.

**Figure 1:**
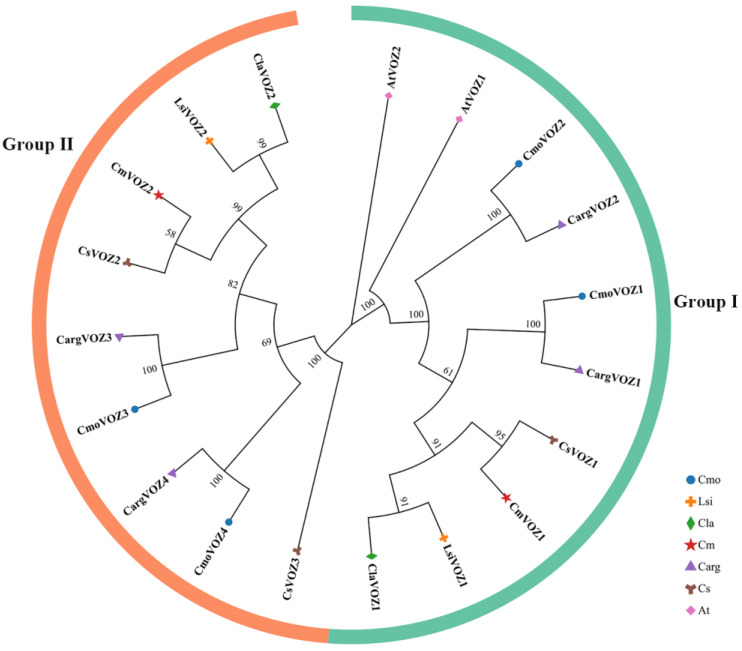
Phylogenetic relationships of VOZ proteins in winter squash (Cmo), cucumber (Cs), melon (Cm), bottle gourd (Lsi), silver-seed gourd (Carg), watermelon (Cla), and *Arabidopsis* (At). All VOZ proteins in the seven species were divided into two groups, with the I and II groups shown in green and orange, respectively. The phylogenetic tree included 2 AtVOZ, 4 CmoVOZ, 3 CsVOZ, 2 CmVOZ, 2 LsiVOZ, 4 CargVOZ, and 2 ClaVOZ proteins.

To elucidate the evolutionary relationships among VOZ proteins, a phylogenetic tree was generated utilizing MEGA11 (v11). The study encompassed a total of 19 VOZ members, comprising 2 from *A. thaliana* and 17 from six Cucurbitaceae species. The resulting phylogenetic tree, depicted in Figure 1, delineates two distinctive groups based on gene structures: Group I, comprising 10 VOZ members, and Group II, encompassing 9 VOZ members. Notably, a close evolutionary association is evident between specific pairs, namely bottle gourd with watermelon, silver-seed gourd with winter squash, and cucumber with melon.

### 3.2. Chromosomal Localization of VOZ Gene Members in Cucurbitaceae

**Figure 2:**
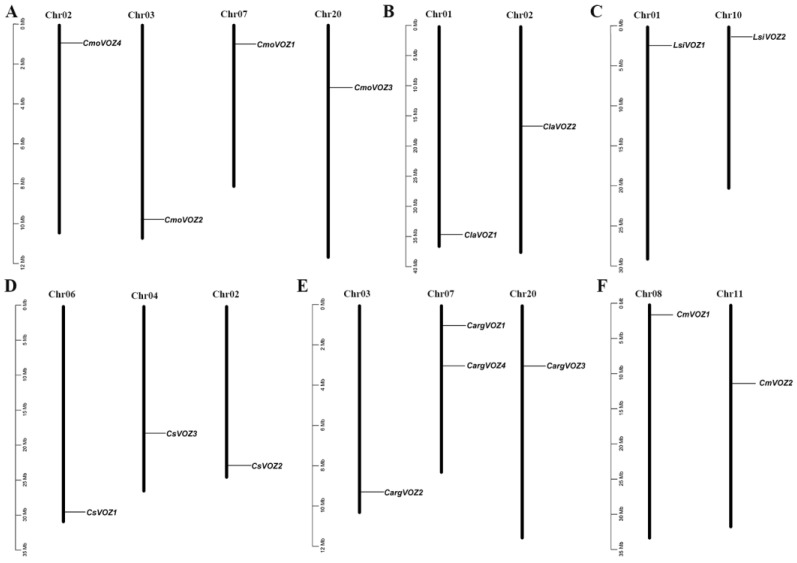
Mapping of the *VOZ* genes on the chromosomes of six Cucurbitaceae species. (**A**) *CmoVOZ* genes in the chromosome of winter body squash. (**B**) *ClaVOZ* gene in the chromosome of watermelon. (**C**) *LsiVOZ* gene in the chromosome of gourd. (**D**) *CsVOZ* gene in the chromosome of cucumber. (**E**) *CargVOZ* gene in the chromosome of silver-seed pumpkin. (**F**) *CmVOZ* gene in the chromosome of melon. The number of chromosomes is marked at the top of each chromosome. The left scale is the length of the chromosome, and the reported scale is in megabases.

The chromosomal localization of 17 *VOZ* genes within the Cucurbitaceae was conducted using TBtools software (v2.061). Figure 2 illustrates the distribution, wherein the four *CmoVOZ* genes from *C. moschata* are situated across four distinct chromosomes (Figure 2A). Similarly, the chromosomal localization of *VOZ* genes in *C. moschata*, *L. siceraria*, and *C. melo* reveals a distribution pattern across two chromosomes, with one gene allocated to each (Figure 2B,C,F). Moreover, the *VOZ* genes in *C. argyrosperma* and *C. sativus* are positioned on three chromosomes, notably featuring two *CargVOZ* genes on chromosome 07 (Figure 2D,E).

### 3.3. Analysis of Structures and Conserved Motifs

**Figure 3:**
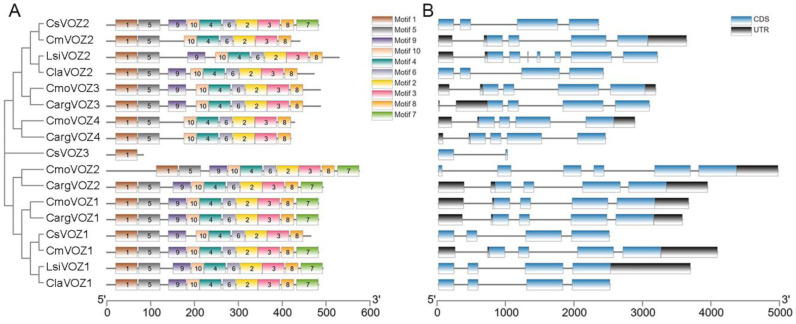
The conserved motifs and gene structures of VOZs. (**A**) The conserved motifs of VOZ proteins. The distribution of these motifs was identified by MEME, and boxes of different colors represent different motifs. The consensus sequences and the amino acid lengths of these motifs were also listed. (**B**) The gene structures of *VOZ* genes.

The examination of exon–intron organization offers valuable insights into the evolutionary dynamics of a gene family. In this study, the structural characteristics of the *VOZ* gene family, encompassing CDS, an untranslated region (UTR), and introns, were delineated through the utilization of TBtools software (v2.061). As depicted in Figure 3B, family members exhibited a range of 2 to 9 exons, accompanied by a minimum of 1 intron and a maximum of 8 introns. Notably, a subset of 8 *VOZ* genes featured 6 introns, while another set of 4 *VOZ* genes displayed 3 introns, revealing shared exon–intron boundaries within these respective groups.

The amino acid conserved motifs of all 17 VOZ proteins were analyzed using the MEME website; the consensus sequences of these motifs are shown in Figure S1. It was found that all VOZ proteins contain motif 1, but CsVOZ3 contains only motif 1 and no other motifs. As shown in Figure 3A, besides CsVOZ3, all the remaining 16 *VOZ* genes contain motif 2–6, motif 8, and motif 10. The vast majority of proteins contain motif 9, except CsVOZ3, CmVOZ2, CmoVOZ4, and CargVOZ4. It is noteworthy that motif 7 appeared in the first group, and in the second group, only CsVOZ2 had motif 7. Furthermore, the phylogenetic tree revealed that evolutionary closely related VOZ proteins exhibit similar motif composition.

### 3.4. Cis-Acting Elements Analysis

**Figure 4:**
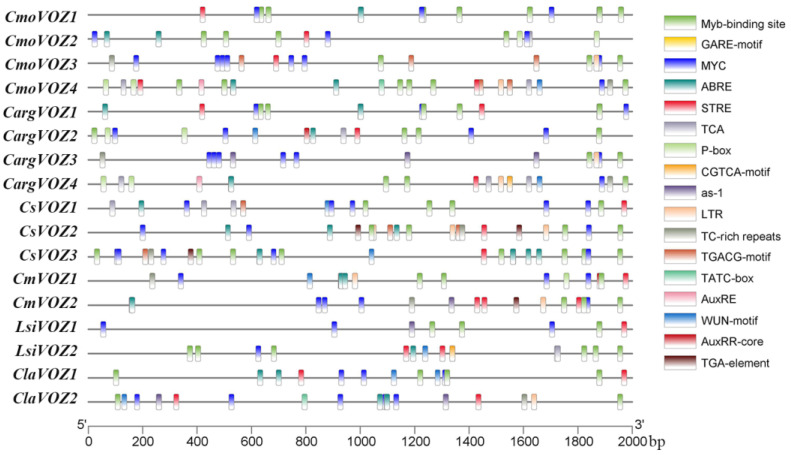
Analysis of *cis*-acting elements in the promoter region of the *VOZ* gene. The names of the *VOZ* genes of silver-seed gourd (*CargVOZ*), watermelon (*CliVOZ*), cucumber (*CsVOZ*), winter squash (*CmoVOZ*), bottle gourd (*LsiVOZ*), and melon (*CmVOZ*) are listed on the left. Different color boxes represented various types of *cis*-elements.

The anticipation of *cis*-acting elements assumes a pivotal role in guiding investigations into the functional involvement of genes in plant development and their responses to diverse abiotic stresses. Within the scope of this study, an exhaustive analysis of *cis*-acting elements in the promoter region of the *VOZ* gene was conducted, using the PlantCARE database. The nine delineated categories encompassed elements responsive to MeJA, gibberellin, abscisic acid, auxin, salicylic acid, drought, low temperature, and wounding, as well as defense and stress-responsive elements. As illustrated in Figure 4, the promoters of *VOZ* genes exhibit a rich assortment of stress-related and phytohormone-responsive elements. Substantial variations in the types and quantities of response elements were discerned among the six species, with *C. moschata* displaying the highest abundance (86), succeeded by *C. sativus* (77) and *C. argyrosperma* (72), while the remaining three species manifested comparatively fewer elements (40 each). Notably, a preponderance of 203 defense and stress-responsive elements, including long terminal repeats (LTRs), TC-rich repeats, MYB, and STRE, was observed, whereas Auxin-responsive elements (AuxRR-core, AuxRE, and TGA-element) exhibited the lowest frequency, totaling eight occurrences. ABA-responsive elements (ABREs) were identified in 41 instances, MeJA-responsive elements (CGTCA-motif and TGACG-motif) occurred 38 times, SA-responsive elements (as-1 and TCA-element) were present in 31 instances, GA-responsive elements (TATC-box, P-box, and GARE-motif) were observed in 14 occurrences, and wound-responsive elements were noted in 10 instances. This comprehensive analysis suggests the potential involvement of the *VOZ* gene family in both plant growth and stress responses.

### 3.5. Expression Analysis of CmoVOZ Gene Family in C. moschata

**Figure 5:**
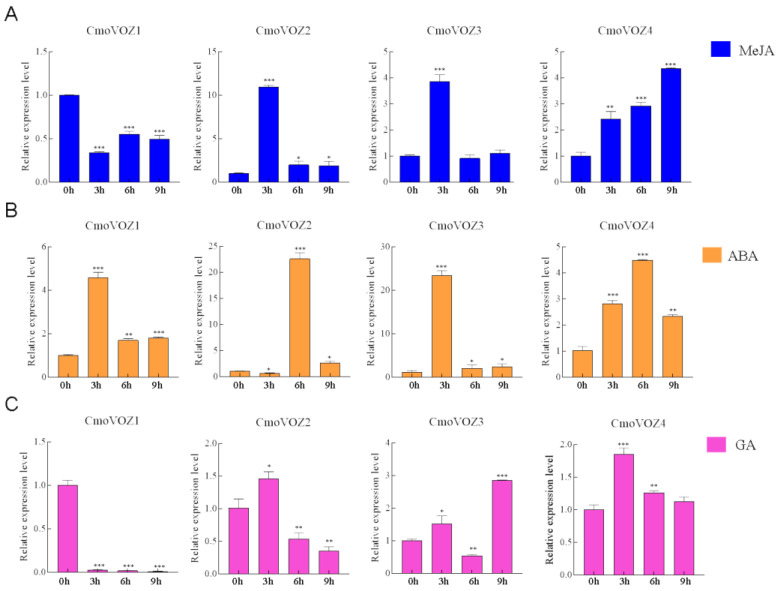
Relative expression levels of the *CmoVOZs* under (**A**) MeJA, (**B**) ABA, and (**C**) GA treatments. Error bars indicate standard deviation of triplicates, and asterisks indicate significant differences (Student’s *t*-test; * *p* < 0.05; ** *p* < 0.01; *** *p* < 0.001).

The significance of the *VOZ* gene family within hormonal pathways prompted an investigation into the expression levels of *CmoVOZ* under the influence of three distinct hormones. As the results showed that the expression of *CmoVOZ1*, *CmoVOZ2*, *CmoVOZ3*, and *CmoVOZ4* was significantly up-regulated in leaves after 100 μM MeJA treatment, *CmoVOZ1*, with the lowest expression at 3 h, increased, but its expression significantly decreased at 6 h and 9 h. The expression of *CmoVOZ2*, *CmoVOZ3*, and *CmoVOZ4* was significantly upregulated, with the highest expression of *CmoVOZ2* and *CmoVOZ3* occurring at 3 h and *CmoVOZ4* at 9 h (Figure 5A). After 100 μM ABA treatment, the highest expression of *CmoVOZ1* and *CmoVOZ3* occurred at 3 h and *CmoVOZ2* and *CmoVOZ4* occurred at 6 h (Figure 5B). After 100 μM GA treatment, *CmoVOZ1* expression was downregulated, and the expression of *CmoVOZ2* and *CmoVOZ4* was maximized at 3 h. *CmoVOZ2* exhibited inhibition at both the 6 h and 9 h time points, while *CmoVOZ4* demonstrated inhibition specifically after 9 h of treatment, as illustrated in Figure 5C.

**Figure 6:**
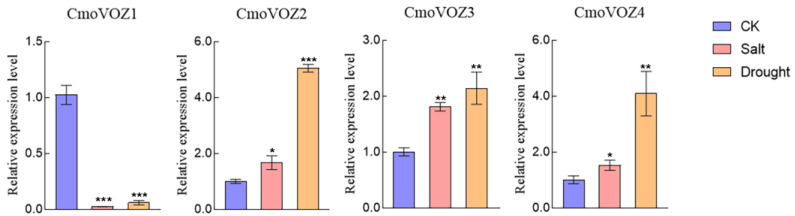
The expression analysis of *CmoVOZs* under salt and drought stress treatments. Error bars indicate the standard deviation of triplicates, and asterisks indicate significant differences (Student’s *t*-test; * *p* < 0.05; ** *p* < 0.01; *** *p* < 0.001).

As a multifaceted regulator, *VOZ* exhibits responsiveness to both biotic and abiotic stresses. To comprehensively explore its adaptive capacity to diverse abiotic stresses, this study evaluated the expression levels of the *CmoVOZ* gene under conditions of drought and salt stress. As depicted in Figure 6, the expression of *CmoVOZ1* was observed to be downregulated under both salt (150 mM NaCl) and drought (20% PEG6000) stresses. In contrast, the genes *CmoVOZ2*, *CmoVOZ3*, and *CmoVOZ4* exhibited significant upregulation under both stress conditions. Specifically, these three genes displayed a range of upregulation from 1.36- to 1.81-fold under salt stress and 2.14- to 4.10-fold under drought stress. In summary, the induced or suppressed expression of the *VOZ* gene family under various abiotic stresses highlights the positive responsiveness of *CmoVOZ2*, *CmoVOZ3*, and *CmoVOZ4* genes to both salt and drought stress.

### 3.6. Transactivation Assay and Subcellular Localization of ComVOZ2

**Figure 7:**
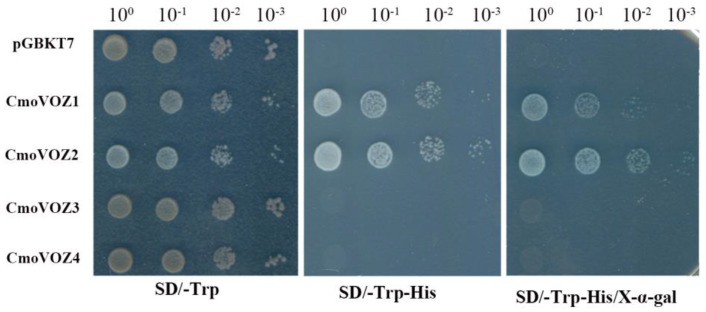
Transactivation assays of four CmoVOZ proteins in yeast cells. The transformed yeast was grown on SD/-Trp media or SD/-Trp-His media. LacZ activity was assessed by β-galactosidase filter lift assay. Empty vector pGBKT7 was used as a negative control.

To ascertain the presence of transcriptional activity in the CmoVOZ proteins, experiments were conducted employing yeast cells, with the pGBKT7 vector serving as a negative control. All yeast cells exhibited normal growth on a standard medium. However, on the selective SD/-Trp-His medium, only yeast cells expressing CmoVOZ1 and CmoVOZ2 grew well and manifested β-galactosidase activity (Figure 7). In contrast, CmoVOZ3- and CmoVOZ4-expressing yeast cells exhibited impaired growth on the SD/-Trp-His medium. These findings imply that CmoVOZ1 and CmoVOZ2 exhibit transcriptional activity in yeast cells, while CmoVOZ3 and CmoVOZ4 do not. This disparity could potentially be attributed to the presence of transcriptional repressor structural domains within full-length transcription factors.

**Figure 8:**
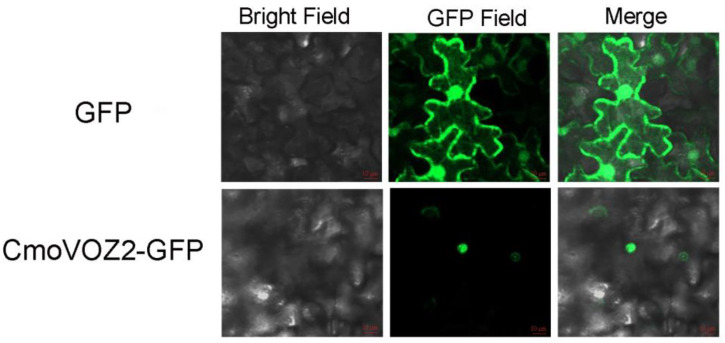
Subcellular localization of CmoVOZ2 during transient expression of *Nicotiana bentamiana*. From left to right, bright field, GFP fluorescent field, and overlay of the channel. The empty GFP vector is located in the cell membrane, nucleus, and cytoplasm, and the fusion protein (CmoVOZ2) is located in the nucleus.

As shown in Figure 8, the fluorescence signal of empty GFP was evident in both the nucleus and the cell membrane of *N. benthamiana* epidermal cells. The CmoVOZ2-GFP fluorescence signal was specifically localized within the nucleus of *N. benthamiana* epidermal cells. This localization pattern suggests that CmoVOZ2-GFP is confined to the nucleus, implying its role as a transcription factor in this cellular compartment.

### 3.7. Analysis of Salt and Drought Stress Tolerance of CmoVOZ2 Gene in Yeast

**Figure 9:**
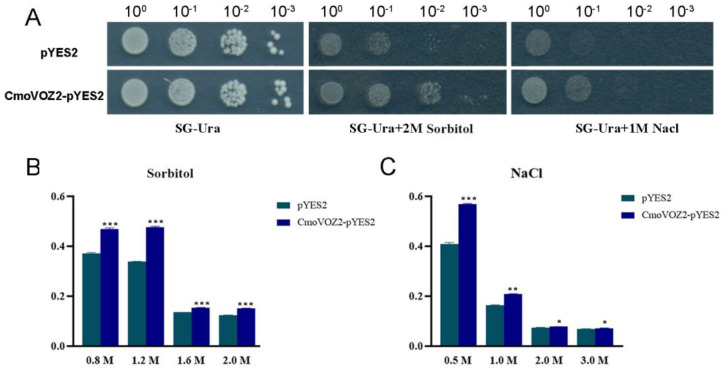
Growth activity of INVSC1 (pYES2) and INVSC1 (pYES2-CmoVOZ2) under different treatments. (**A**) The growth of pYE 2 and pYES2-CmoVOZ2 yeast cells under sorbitol and NaCl treatments. The growth rates of yeast cells after (**B**) drought and (**C**) salt stress. The yeast cells were adjusted to an equal cell density and then cultured in NaCl (0.5 M, 1.0 M, 2.0 M, and 3.0 M) and sorbitol (0.8 M, 1.2 M, 1.6 M, and 2.0 M) at 28 °C with shaking for 24 h (*t*-test analysis, * *p* < 0.05, ** *p* < 0.01, *** *p* < 0.001).

To further investigate the role played by VOZ in stress response, we exposed yeast cells harboring both the pYES2 plasmid (control group) and the pYES2-CmoVOZ2 plasmids to stress conditions involving NaCl and sorbitol treatments. The findings indicate that yeast cells containing either the empty pYES2 or the pYES2-CmoVOZ2 plasmids exhibited growth on solid SG-Ura medium under normal conditions (Figure 9). However, after treatment with sodium chloride and sorbitol, there were obvious differences. Nevertheless, discernible distinctions became apparent following treatment with sodium chloride and sorbitol. In the presence of sorbitol, yeast cells harboring the pYES2-CmoVOZ2 plasmid exhibited a higher growth rate compared to the control yeast cells. This observation suggests that CmoVOZ2 imparts enhanced drought tolerance to transgenic yeast cells. Similarly, in the presence of sodium chloride, yeast cells with the pYES2-CmoVOZ2 plasmid displayed a higher growth rate compared to the control yeast cells. This observation indicates that CmoVOZ2 imparts elevated salt tolerance to transgenic yeast cells.

Furthermore, an assessment of the growth status of yeast cells was conducted following 24 h of exposure to NaCl and sorbitol treatments. The findings revealed an ameliorated growth status of yeast cells expressing VOZ under both sodium chloride and sorbitol treatments. This observation implies that yeast cells transformed with the *VOZ* gene exhibit heightened tolerance to these abiotic stresses. Thus, these results collectively indicate that the expression of the *CmoVOZ2* gene in yeast enhances its tolerance to drought and salt treatments.

## 4. Discussion

Plants undergo a spectrum of biotic and abiotic stresses throughout their growth and developmental stages, leading to significant reductions in crop yield and quality, and potentially culminating in fatality [1]. For evolution, plants have evolved an array of physiological mechanisms to proficiently counteract such stresses. Transcription factors, crucial regulators in plant growth and resistance, exert control over chromatin and transcription processes by discerning specific nucleotide sequences [4,7]. This ability enables them to guide genome expression within intricate systems. VOZ proteins represent a plant-specific subclass of transcription factors with pivotal functions in the modulation of plant growth and development, particularly in the realm of VOZ-mediated responses to abiotic stress [10,11,12]. The *VOZ* gene family has been reported in a variety of plant species [11,12,13,15], but there is still a lack of comprehensive studies on the function of *VOZ* genes in cucurbits and their response to growth, development, and abiotic stresses. Consequently, there exists a pressing need for an in-depth investigation of VOZ in cucurbits.

In our study, a total of 17 VOZ proteins were discerned within the Cucurbitaceae family. Winter squash, silver-seed gourd, melon, bottle gourd, watermelon, and cucumber exhibited the presence of 4, 2, 2, 2, 4, and 3 VOZ protein sequences, respectively. We conducted an analysis encompassing amino acid composition, Mw, and pI across diverse VOZ members in cucurbits, *Arabidopsis*, rice, soybean, and quinoa (Table 1).

Notably, the size of the *VOZ* gene family in winter squash, watermelon, and cucumber surpasses that found in silver-seeded gourd, melon, bottle gourd, *S. lycopersicum*, *A. thaliana*, and rice, each containing two members, but remains below that of soybean, which harbors six members. The prevalence of transcription factors in the course of genome evolution has been documented to heavily rely on gene sequence duplication (see relevant literature on single gene duplication). Disparities in the number of *VOZ* genes among distinct plant species may be attributed to genome duplication events, leading to the deletion or amplification of *VOZ* genes within Cucurbitaceae [40].

In addition, VOZ proteins from different species differed in the number of amino acids, Mw, and pI. In di-cotyledonous plants such as *A. thaliana*, soybean, and tomato, the molecular weights of VOZ proteins ranged from 50.57 to 54.02 kDa, with pI values spanning from 5.17 to 6.21. In monocotyledonous plants like rice and quinoa, VOZ proteins exhibited molecular weights ranging from 45.53 to 69.90 kDa, along with pI values ranging from 5.10 to 7.56. The majority of VOZ proteins identified within the Cucurbitaceae family displayed Mw within the range of 47 to 64.76 kDa and pI values ranging from 5.17 to 6.44. These findings suggest a closer resemblance between the characteristics of most VOZ proteins in Cucurbitaceae and those found in dicotyledonous *A. thaliana*, indicating a probable evolutionary conservation of the dual *VOZ* genes.

The genomic distribution analysis revealed that the identified 17 *VOZ* genes in Cucurbitaceae are distributed across 16 chromosomes, as illustrated in Figure 2. Specifically, the four *CmoVOZ* genes were mapped to distinct chromosomes. Additionally, the genomic locations of *CargVOZ* in silver-seed gourd and *CsVOZ* genes in cucumber were distributed across three chromosomes, with two *CargVOZ* genes located on chromosome 07. Furthermore, the *VOZ* genes identified in melon, bottle gourd, and watermelon were uniformly localized on two chromosomes, with one gene per chromosome. The structural diversity in exon/intron arrangements holds significant importance in the evolutionary dynamics of plant gene families [41,42]. As depicted in Figure 3, the family members exhibited a range of 2 to 9 exons and 1 to 8 introns. Notably, eight *VOZ* genes, characterized by six introns, and four *VOZ* genes, characterized by three introns, displayed analogous exon–intron boundaries. The exon–intron architecture of family members varied between 2 and 9 exons and 1 and 8 introns. Additionally, a consistent structural pattern was observed within a species, exemplified by members of the *CmoVOZ* and *CmVOZ* gene families featuring seven exons, while the *ClaVOZ* gene family members contained four exons. The number of UTR of *VOZ* genes also varies across species, 1–3 UTRs in winter squash, 1 and 2 UTRs in bottle gourd, 2 and 3 UTRs in melon, respectively, 2–3 UTRs in silver-seed gourd, and no UTR in watermelon and cucumber.

Various members of the *VOZ* family encompassed highly conserved domains, potentially associated with their analogous and identical regulatory functions, as reported in prior research [12]. The present study additionally identified 10 conserved motifs to facilitate a more in-depth investigation into the evolutionary aspects of 17 VOZ proteins. Remarkably, all VOZ proteins universally feature motif 1, whereas CsVOZ3 exclusively incorporates motif 1 without any additional motifs. Aside from CsVOZ3, the remaining 16 VOZ proteins encompass motifs 2–6, motif 8, and motif 10, indicating a high degree of conservation and potential functional significance in the *VOZ* gene family. Furthermore, motif 7 is evident in the first group, with CsVOZ2 being the sole member in the second group possessing motif 7. These findings underscore the observation that evolutionarily closely related VOZ proteins share a similar motif composition. The spatial distribution and quantity of motifs within the identical branch exhibited a notable resemblance. For example, both LsiVOZ1 and ClaVOZ1 contain 10 motifs and LsiVOZ2 and ClaVOZ2 contain 9 motifs, both CmoVOZ1-2 and CargVOZ1-2 contain 10 motifs, both CmoVOZ3 and CargVOZ3 contain 9 motifs, and both CmoVOZ4 and CargVOZ4 contain 8 motifs; and both LsiVOZ1 and ClaVOZ1 contain 10 motifs, and both LsiVOZ2 and ClaVOZ2 contain 9 motifs. The close evolutionary association between silver-seed gourd and *C. moschata* was congruent with the findings derived from the phylogenetic tree analysis.

Promoters constitute pivotal repositories of cis-acting elements crucial for gene initiation and transcriptional regulation. The *VOZ* gene family assumes a pivotal role in diverse stress responses [16,19]. The anticipation of cis-acting elements holds the potential to guide investigations into the responsive effects of *VOZ* genes to a spectrum of abiotic stress. This study involved the prediction of numerous cis-acting elements within the Cucurbitaceae (Figure 4), revealing a preponderance of defense and stress-responsive elements, with a subsequent prominence of ABA-responsive elements, while Auxin-responsive elements were comparatively less abundant. These outcomes suggest that the expression of *VOZ* genes primarily hinges on regulation by defense and stress-related factors, with the additional involvement of ABA, JA, GA, and other diverse cis-acting elements.

*VOZ* genes are presumed to exhibit responsiveness to dynamic environmental conditions, playing a role in both the modulation of plant growth and involvement in developmental processes [11,14,15,18]. The pumpkin plants were subjected to two abiotic stresses and three hormonal treatments to examine the expression level of the *VOZ* gene under stress. As depicted in Figure 5, the regulatory impact on the *CmoVOZ* gene was notably pronounced under MeJA, ABA, and GA treatments. Specifically, *CmoVOZ1* demonstrated significant downregulation under MeJA and GA treatment, coupled with a substantial upregulation under ABA treatment. Conversely, *CmoVOZ2* exhibited significant upregulation under ABA and GA treatment, with an initial upregulation at 3 h followed by a decline at 6 h under GA treatment. Both *CmoVOZ3* and *CmoVOZ4* experienced significant upregulation under all three hormonal treatments, except *CmoVOZ3* downregulation at 6 h in response to GA treatment. Under salt and drought treatments, *CmoVOZ1* demonstrated significant inhibition, *CmoVOZ2* and *CmoVOZ4* exhibited substantial upregulation, and no significant alteration was observed in CmoVOZ3 expression. Notably, the diverse responses of different *CmoVOZ* genes to stress conditions suggest their varied functional roles in stress response mechanisms.

The transcriptional activity experiments conducted on the CmoVOZ protein family revealed that yeast cells expressing CmoVOZ1 and CmoVOZ2 thrived on SD/-Trp-His medium, exhibited β-galactosidase activity, and demonstrated transcriptional competence. Conversely, yeast cells expressing CmoVOZ3 and CmoVOZ4 exhibited impaired growth on SD/-Trp-His medium and lacked transcriptional activity. It was shown that CmoVOZ1 and CmoVOZ2 are the major members of the pumpkin *VOZ* gene family exerting their functions. Moreover, the outcomes of the subcellular localization analysis demonstrated that the CmoVOZ2 protein was predominantly localized within the nucleus, as illustrated in Figure 8. This additional evidence supports the notion that CmoVOZ2 proteins have the potential to interact with other nuclear proteins or directly bind to nucleic acids, thereby orchestrating the regulation of target genes and playing a significant role in stress-response pathways. To delve deeper into the functional implications of the *CmoVOZ* gene in abiotic stress contexts, we conducted simulations of drought and salt stress (Figure 9). The findings revealed that the growth rate of yeast cells harboring pYES2-CmoVOZ2 in the presence of sorbitol and sodium chloride surpassed that of the control yeast (pYES2). This observation suggests the active involvement of VOZ2 in stress response mechanisms, thereby enhancing the salt and drought tolerance of yeast cells. Derived from these findings, it is deduced that the *CmoVOZ* gene potentially assumes a pivotal role in the growth and abiotic stress of pumpkin.

## 5. Conclusions

In this study, 17 *VOZ* genes were identified in the Cucurbit genome using bioinformatics methods. These genes were evenly distributed on 16 chromosomes and phylogenetically divided into two subfamilies. Comprehensive studies on their basic characteristics, genomic locations, gene structures, and conserved motifs have laid a solid foundation for elucidating the evolutionary relationships within the *VOZ* gene family. The analysis of gene expression under hormonal and abiotic stresses revealed the potential regulatory role of *VOZs* in plant growth and development. In addition, their involvement in the mechanism of salt stress was observed. These findings lay the foundation for further exploration of the biological functions of the *VOZ* family in *C. moschata*.

## Figures and Tables

**Table 1:** The information on *VOZ* genes identified in six Cucurbitaceae species and a summary of different members of the *VOZ* gene family reported in the literature.

Taxon (Species)	Ploidy	N ^a^	N ^b^	Gene Name	Gene ID	CDS (bp)	aa	Mw (kDa)	pI	Ref.
*C. argyrosperma*	diploid	10	4	*CargVOZ1*	Carg07755	1452	483	54.03	5.88	
				*CargVOZ2*	Carg15320	1482	493	55.20	6.22	
				*CargVOZ3*	Carg03109	1464	487	54.47	5.67	
				*CargVOZ4*	Carg05122	1287	420	47.01	5.17	
*C. lanatus*	diploid	11	2	*ClaVOZ1*	Cla97C01G023230.1	1452	483	53.95	5.87	
				*ClaVOZ2*	Cla97C02G036280.1	1422	473	52.94	5.83	
*C. melo*	diploid	12	2	*CmVOZ1*	MELO3C007248.1	1452	483	54.06	6.04	
				*CmVOZ2*	MELO3C019321.1	1404	440	49.23	6.44	
*C. moschata*	allotetraploid	20	4	*CmoVOZ1*	CmoCh07G002120.1	1452	483	54.03	5.88	
				*CmoVOZ2*	CmoCh03G012970.1	1452	576	64.76	6.42	
				*CmoVOZ3*	CmoCh20G006520.1	1464	487	54.49	5.64	
				*CmoVOZ4*	CmoCh02G001990.1	1287	428	47.83	5.18	
*C. sativus*	diploid	7	3	*CsVOZ1*	CsaV3_6G050500.1	1452	483	53.98	6.04	
				*CsVOZ2*	CsaV3_2G034550.1	1398	465	52.20	5.69	
				*CsVOZ3*	CsaV3_4G028820.1	252	83	9.52	9.69	
*L. siceraria*	diploid	11	2	*LsiVOZ1*	Lsi01G002760.1	1482	493	55.07	5.88	
				*LsiVOZ2*	Lsi10G000870.1	1593	530	59.30	5.76	
*A. thaliana*	diploid	5	2	*AtVOZ1*	At1g28520	1461	486	54.08	5.73	[9,16,17]
				*AtVOZ2*	At2g42400	1353	450	50.57	5.17	
*Oryza sativa*	diploid	12	2	*OsVOZ1*	LOC_Os01g54930	1290	429	48.11	5.10	[15]
				*OsVOZ2*	LOC_Os05g43950	1926	641	69.90	5.39	
*Glycine max*	tetraploid	9	6	*GmVOZ1A*	Glyma.07G202700	1434	477	53.12	5.87	[11]
				*GmVOZ1C*	Glyma.13G172900	1437	478	53.35	5.71	
				*GmVOZ1E*	Glyma.11G211900	1380	459	51.16	5.83	
				*GmVOZ1G*	Glyma.10G068900	1398	465	51.63	6.21	
				*GmVOZ2B*	Glyma.06G285800	1437	478	54.02	5.56	
				*GmVOZ2D*	Glyma.12G120100	1440	479	53.85	5.26	
*C. quinoa*	allotetraploid	9	4	*CqVOZ1*	AUR62014889	1455	484	54.31	5.70	[12]
				*CqVOZ2*	AUR62024758	1569	522	58.08	5.48	
				*CqVOZ3*	AUR62031095	1230	409	45.93	7.56	
				*CqVOZ4*	AUR62037692	1566	521	57.93	5.49	
*S. lycopersicum*	diploid	12	2	*SIVOZ1*	Solyc02g077450	1404	467	52.08	5.26	[13]
				*SIVOZ2*	Solyc10g008880	1434	477	53.54	6.00	

**a**: number of chromosomes in a haploid set; **b**: number of VOZ genes in a genome.

## Data Availability

All data are displayed in the manuscript and Supplementary Materials.

## Supplementary Materials

The following supporting information can be downloaded at: https://www.mdpi.com/article/10.3390/genes15030307/s1, Table S1: Chromosomal information of VOZ genes in six Cucurbitaceae species. Table S2: All primers used for gene cloned, qRT-PCR, and vector construction. Figure S1: The consensus sequences of ten motifs.
