# Supplementary material for: Genome-Wide Identification of Vascular Plant One-Zinc-Finger Gene Family in Six Cucurbitaceae Species and the Role of CmoVOZ2 in Salt and Drought Stress Tolerance
Source: Genes (Basel). 2024 Feb 27;15(3):307. doi: 10.3390/genes15030307 (PMC10969924; doi:10.3390/genes15030307)
Supplement: Supplementary file 1 [file genes-15-00307-s001.zip › genes-2869058-supplementary.pdf]

Supplementary Materials

Genome-Wide Identification of *VOZ* Gene Family in Six Cucurbitaceae Species and the Role of *CmoVOZ2* in Salt and Drought Stress Tolerance

Minyan Xu †, Zhi Zhang †, Yuhuan Jiao, Yaling Tu and Xin Zhang *

National Engineering Laboratory of Crop Stress Resistance Breeding, School of Life Sciences, Anhui Agricultural University, Hefei 230036, China
* Correspondence: xinzhang@ahau.edu.cn
† These authors contributed equally to this work.

Table S1. Chromosomal information of *VOZ* genes in six Cucurbitaceae species

Name	Gene ID	Chromosome	Location
CargVOZ1	Carg07755	Chr07	56865 .. 60425 (-)
CargVOZ2	Carg15320	Chr03	338531 .. 342459 (+)
CargVOZ3	Carg03109	Chr20	134324 .. 137402 (+)
CargVOZ4	Carg05122	Chr07	12737 .. 15175 (-)
ClaVOZ1	Cla97C01G023230.1	Chr01	34763205 .. 34765707 (+)
ClaVOZ2	Cla97C02G036280.1	Chr02	16754161 .. 16756569 (-)
CmVOZ1	MELO3C007248.1	Chr08	1709815 .. 1713888 (-)
CmVOZ2	MELO3C019321.1	Chr11	11404847 .. 11407858 (-)
CmoVOZ1	CmoCh07G002120.1	Chr07	1063694 .. 1067347 (-)
CmoVOZ2	CmoCh03G012970.1	Chr03	9786940 .. 9791897 (+)
CmoVOZ3	CmoCh20G006520.1	Chr20	3187678 .. 3190850 (-)
CmoVOZ4	CmoCh02G001990.1	Chr02	932111 .. 934978 (-)
CsVOZ1	CsaV3_6G050500.1	Chr06	29459015 .. 29470420 (+)
CsVOZ2	CsaV3_2G034550.1	Chr02	22956134 .. 22960364 (+)
CsVOZ3	CsaV3_4G028820.1	Chr04	18302368 .. 18304592 (-)
LsiVOZ1	Lsi01G002760.1	Chr01	2456480 .. 2460158 (-)
LsiVOZ2	Lsi10G000870.1	Chr10	1457206 .. 1460401 (+)

Table S2. All primers used for gene cloned, qRT-PCR, and vector construction

Primer name	Forward Primer Sequence (5′–3′)	Reverse Primer Sequence (5′–3′)
CmoVOZ1 (gene cloning)	TCTATTTTCTCGCTCCTAC	AACCAGCTTTACCGTTATC
CmoVOZ2 (gene cloning)	TCCCCGGGTCTAGAAGA	AGAAAAAAAGAGTTATTTATGT
CmoVOZ3 (gene cloning)	TTTACCCTGATTTTCGACCC	GGTCGGTTGATCAGTTAGTG
CmoVOZ4 (gene cloning)	TCATTACGCTCATTCCTAAA	GACGAGTATGTACCATCATAACAAT
Cmo-β-Actin (qRT-PCR)	GTGCCTGCTATGTATGTTGCC	GGTCCAAACGGAGAATGGCATG
CmoVOZ1 (qRT-PCR)	TTAGTACGAAGGCACAAGGA	CACTATAATCCGGCAATGAC
CmoVOZ2 (qRT-PCR)	GAGTCATGCCACCCAATGGA	CGATTGAAACCATCAGCCTGC
CmoVOZ3 (qRT-PCR)	CATGGCTACTCAGCCTAATTGTCAA	AATGCATGGCAATCCAAAGT
CmoVOZ4 (qRT-PCR)	TTATGCAGAACTTAGTGGTC	TGTCCTGACTAAAGTCTCCC
CmoVOZ2 (1305-GFP)	ggacagcccagatcaactagtATGGGAAAACATTCGAAGACCA	ggtcctcgagacgtctctagaTGTAAGGTAATATTCGCTCAAATTCTC
CmoVOZ2 (pYES2)	cgataaggtacctaaggatccATGGGAAAACATTCGAAGACCA	tgctggatatctgcagaattcTGTAAGGTAATATTCGCTCAAATTCT
CmoVOZ1 (pGBKT7)	atggccatggaggccgaattcATGGGGAAGCATTCGAAGACC	ctagttatgcggccgctgcagCTACGTAAGGTAATATTCGCTCAAATT
CmoVOZ2 (pGBKT7)	atggccatggaggccgaattcATGGGAAAACATTCGAAGACCA	ctagttatgcggccgctgcagTGTAAGGTAATATTCGCTCAAATTCT
CmoVOZ3 (pGBKT7)	atggccatggaggccgaattcATGCCGAAGGGTTTGAAGAGT	ctagttatgcggccgctgcagTCAGTTAGTGTTAGACATATTCTGATTATGTG
CmoVOZ4 (pGBKT7)	atggccatggaggccgaattcATGCCGAGGGGTTTGAAGA	ctagttatgcggccgctgcagTTATTTGACTGATTGTCCAAGCTCC

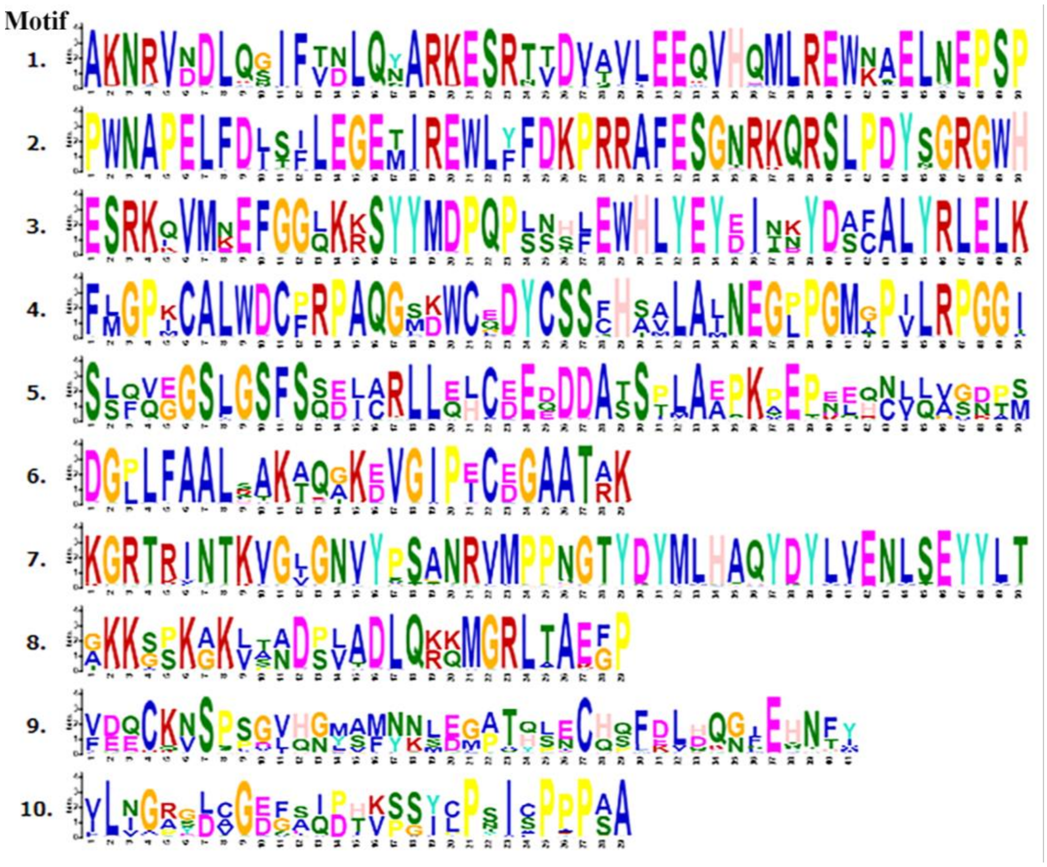

Figure S1. The consensus sequences of ten motifs.
